# Alignment of short reads to multiple genomes using hashing

**DOI:** 10.1186/1471-2105-15-S10-P23

**Published:** 2014-09-29

**Authors:** Quang Tran, Vinhthuy Phan

**Affiliations:** 1 Bioinformatics Program, University of Memphis, Memphis, TN 38152 USA; 2 Department of Computer Science, University of Memphis, Memphis, TN 38152, USA

## Background

Recent advances in biotechnology have enabled high-throughput sequencing of genomes based on large numbers of short reads. Current methods [1,2], however, depend mostly on aligning reads to only one reference genome at a time, making it difficult to differentiate sequencing errors from single nucleotide variants (SNV).

## Materials and methods

Inspired by [3], we propose a new method that attempts to take advantage of multiple genomes and SNV information to align reads. This approach is promising in that it allows us to distinguish between sequencing errors and SNV. Our proposed alignment algorithm uses read fragments to identify seeds and extend these seeds to find occurrences of reads in the genome. In this study, we have developed and implemented an algorithm using multiple genomes that captures genomic variations, indexes the multiple genomes and operates short read alignment on a collection of genomes. The preliminary result was validated on *Aspergillus fumigatus*.

## References

[B1] Gontarz PM Berger J Wong CF SRmapper: a fast and sensitive genome-hashing alignment tool Bioinformatics 2013 29 3 316 321 23267171 10.1093/bioinformatics/bts712

[B2] Langmead B Salzberg SL Fast gapped-read alignment with Bowtie 2 Nat Methods 2012 9 4 357 359 22388286 10.1038/nmeth.1923 PMC3322381

[B3] Huang L Popic V Batzoglou S Short read alignment with populations of genomes Bioinformatics 2013 29 13 i361 i370 23813006 10.1093/bioinformatics/btt215 PMC3694645

